# Identification of *GAA* variants through whole exome sequencing targeted to a cohort of 606 patients with unexplained limb-girdle muscle weakness

**DOI:** 10.1186/s13023-017-0722-1

**Published:** 2017-11-17

**Authors:** Katherine Johnson, Ana Töpf, Marta Bertoli, Lauren Phillips, Kristl G. Claeys, Vidosava Rakocevic Stojanovic, Stojan Perić, Andreas Hahn, Paul Maddison, Ela Akay, Alexandra E. Bastian, Anna Łusakowska, Anna Kostera-Pruszczyk, Monkol Lek, Liwen Xu, Daniel G. MacArthur, Volker Straub

**Affiliations:** 1 John Walton Muscular Dystrophy Research Centre, Institute of Genetic Medicine, Newcastle University, International Centre for Life, Central Parkway, Newcastle upon Tyne, NE1 3BZ UK; 2 Department of Neurology and Institute of Neuropathology, RWTH Aachen University Hospital, Aachen, Germany; 3 Department of Neurology, University Hospitals Leuven, Leuven, Belgium; 4 Laboratory for Muscle Diseases and Neuropathies, Research Group Experimental Neurology, Department of Neurosciences, KU Leuven (University of Leuven), Leuven, Belgium; 5 Neurology Clinic CCS, School of Medicine, University of Belgrade, Belgrade, Serbia; 6 Department of Child Neurology, Justus-Liebig University, Gießen, Germany; 7 Queen’s Medical Centre, Nottingham, UK; 8 Clinical Hospital Colentina, Carol Davila University of Medicine and Pharmacy, Bucharest, Romania; 9 Department of Neurology, Medical University of Warsaw, Warsaw, Poland; 10 Analytic and Translational Genetics Unit, Massachusetts General Hospital, Boston, MA USA; 11 Program in Medical and Population Genetics, Broad Institute of Harvard and MIT, Cambridge, MA USA

**Keywords:** Whole exome sequencing, Sequence variants, Pompe disease

## Abstract

**Background:**

Late-onset Pompe disease is a rare genetic neuromuscular disorder caused by a primary deficiency of α-glucosidase and the associated accumulation of glycogen in lysosomal vacuoles. The deficiency of α-glucosidase can often be detected using an inexpensive and readily accessible dried blood spot test when Pompe disease is suspected. Like several neuromuscular disorders, Pompe disease typically presents with progressive weakness of limb-girdle muscles and respiratory insufficiency. Due to the phenotypic heterogeneity of these disorders, however, it is often difficult for clinicians to reach a diagnosis for patients with Pompe disease. Six hundred and six patients from a European population were recruited onto our study. Inclusion criteria stipulated that index cases must present with limb-girdle weakness or elevated serum creatine kinase activity. Whole exome sequencing with at least 250 ng DNA was completed using an Illumina exome capture and a 38 Mb baited target. A panel of 169 candidate genes for limb-girdle weakness was analysed for disease-causing variants.

**Results:**

A total of 35 variants within *GAA* were detected. Ten distinct variants in eight unrelated index cases (and four siblings not sequenced in our study) were considered disease-causing, with the patients presenting with heterogeneous phenotypes. The eight unrelated individuals were compound heterozygotes for two variants. Six patients carried the intronic splice site c.-13 T > G transversion and two of the six patients also carried the exonic p.Glu176ArgfsTer45 frameshift. Four of the ten variants were novel in their association with Pompe disease.

**Conclusions:**

Here, we highlight the advantage of using whole exome sequencing as a tool for detecting, diagnosing and treating patients with rare, clinically variable genetic disorders.

## Background

Pompe disease (OMIM 232300) is a rare autosomal recessive lysosomal storage disorder that most prominently affects muscle tissue. The disease is generally classified into two broad categories: an infantile- and a late- onset form. Patients with the infantile-onset form of the disease typically present with generalised weakness, hypotonia, respiratory distress and cardiomyopathy, and without intervention do not survive beyond 12 months of age [1]. Late-onset Pompe disease is more clinically heterogeneous [2], yet often displays a central characteristic manifestation of a slowly progressive proximal myopathy. This occurs with respiratory weakness and elevated serum creatine kinase activity, while there is normally no clinically relevant cardiac involvement [3]. Estimates of the frequency of all cases of Pompe disease vary, but have been reported to be as high as approximately 1:40,000 [4, 5]. The late-onset classification of the disease is even rarer at a frequency of 1:57,000 [5].

The varied clinical spectrum can be attributed to the many different genetic mutations that are associated with Pompe disease; to date, the associations of over 522 variants have been reported and collated by the Pompe Disease Mutation Database [6]. Such mutations occur within the *GAA* gene and result in a deficiency of the encoded lysosomal enzyme, acid α-glucosidase, which is essential for glycogen hydrolysis. The accumulation of glycogen within lysosomes subsequently impairs the correct functioning of the organelles and the affected tissue, primarily skeletal and cardiac muscle [7], and results in the clinical presentation of Pompe disease.

Considering the rarity and variability of the disorder, a correct clinical diagnosis is often difficult to achieve, and so many patients are therefore not treated with an efficacious disease-modifying enzyme replacement therapy (ERT) in a timely manner. ERT, a recombinant human acid α-glucosidase termed alglucosidase alfa, has been reported to extend survival in infantile Pompe disease [8] and ameliorate disease progression of the late-onset form [9]. As prompt diagnosis and treatment is beneficial to patient survival [10], a more robust investigatory approach is required to detect and diagnose affected individuals in the early stages of the disease.

Exome sequencing is a useful tool to interrogate the proportion of the genome that is enriched for functional coding variants, specifically those that are able to disrupt protein structure and function [11]. This unbiased analysis enables the detection of distinct mutations in patients with overlapping phenotypes, overall expanding existing genotype-phenotype correlations. Exome sequencing, therefore, has the benefit of furthering the understanding of disease pathology, offering accurate diagnoses where the traditional methodologies of clinical examinations failed to do so, and in some cases also enabling a prompt intervention in the disease progression.

The MYO-SEQ project was established in 2014 and aimed to use whole exome sequencing to (i) contribute to the diagnostic pathway of patients affected by limb-girdle muscular weakness, (ii) improve the diagnostic awareness of rare genetic neuromuscular diseases, and (iii) speed up the integration of next-generation sequencing technologies into healthcare [12]. We screened 606 patients with unexplained limb-girdle weakness for potentially pathogenic variants in 169 genes that are known to be associated with muscle disease. Here, we report on the characterisation of twelve European patients (eight index cases and four siblings) who harboured disease-associated variants within *GAA* as identified through the MYO-SEQ project.

## Methods

### Patient recruitment and inclusion criteria for whole exome sequencing

Ethical approval was granted by the Newcastle and North Tyneside research ethics committee (REC reference number 09/H0906/28) and by the local ethical committees of the participating centres. A standardised form for collecting detailed phenotypic information was created using the PhenoTips online software tool [13]: this was completed by the referring clinician for each patient enrolled onto the project. Informed written consent was given by the patients, who were anonymised by the collaborating centres by using unique MYO-SEQ patient identification codes. The fundamental requirement for inclusion in the project was that of unexplained limb-girdle muscle weakness and/or elevated serum creatine kinase activity.

### Lysis of whole blood cells

Blood samples were taken from each patient using an EDTA Vacutainer® Safety-Lok™ system (BD Biosciences, UK). Two buffers were prepared prior to cell lysis: Buffer A (pH 8.0; 10 mM Tris-HCl, 320 mM sucrose, 5 mM MgCl_2_, 1% Triton X-100) and Buffer B (pH 8.0; 400 mM Tris-HCl, 0.5 M EDTA pH 8.0, 150 mM NaCl, 1% SDS). Five millilitres (ml) of whole blood was mixed with 40 ml of Buffer A for 4 minutes (min) at room temperature before centrifugation at 3000 revolutions per minute (rpm) for 10 min. The cell pellet was resuspended in 20 ml Buffer A before the mixing and centrifugation steps repeated once more. The pellet was resuspended in 2 ml Buffer B, mixed for 10 min at room temperature with 500 μl sodium perchlorate, and incubated at 65 °C for 25 min with regular vortexing.

### Nucleic acid extraction from lysed whole blood cells

The preparation of lysed cells was mixed for 10 min at room temperature with 2 ml ice cold chloroform followed by centrifugation at 4 °C for 10 min at 3000 rpm. The supernatant was mixed by gentle inversion with 6 ml 98% ethanol, and the visible DNA precipitate transferred to 500 μl 70% ethanol. The sample was centrifuged for 1 min at 14,000 rpm and the pelleted DNA precipitate air dried at room temperature. The DNA was dissolved in 300 μl TE buffer (10 mM Tris base, 1 mM EDTA [pH 8.0]) overnight at 4 °C followed by a final 1 h incubation at 54 °C. The nucleic acid was quantified using a NanoDrop™ 8000 spectrophotometer (ThermoScientific, Surrey, UK) and stored at -20 °C.

### Whole exome sequencing

Whole exome sequencing and data processing [14] were performed by the Genomics Platform at the Broad Institute of Harvard and MIT (Broad Institute, Cambridge, MA, USA). Briefly, whole exome sequencing was performed on DNA samples (>250 ng at >2 ng/μl) using Illumina exome capture (38 Mb target). Our exome sequencing pipeline included sample plating, library preparation (2-plexing of samples per hybridisation), hybrid capture, sequencing (76 bp paired reads), sample identification, quality control check, and data storage. Our hybrid selection libraries cover >80% of targets at 20× and an overall mean target coverage of >80×, while *GAA* had mean coverage of 87.1×. The exome sequencing data was de-multiplexed and each sample’s sequence data were aggregated into a single Picard BAM file. The data were processed through a pipeline based on Picard using base quality score recalibration and local alignment at known insertions/deletions. The reads were mapped to the human genome build 37 (hg19) using the Burrows-Wheeler Aligner. Single nucleotide polymorphisms and insertions/deletions were jointly called using the Genome Analysis Toolkit HaplotypeCaller package v3.1 [15–17]. Default filters were applied to the variant calls using the Genome Analysis Toolkit Variant Quality Score Recalibration approach, and the variants were annotated using Variant Effect Predictor.

### Analysis of whole exome sequencing data

The variant call set was uploaded onto the Broad Institute of Harvard and MIT’s *seqr* platform. The biological relevance of the variants identified within *GAA* was determined by considering the (i) population frequency detailed by the Exome Aggregation Consortium (ExAC) of the Broad Institute of Harvard and MIT [14], (ii) deleteriousness of the variant predicted by PolyPhen-2 [18], SIFT [19], MutationTaster2 [20], and FATHMM [21], and (iii) ClinVar reports of pathogenicity [22] and published literature.

### Reporting of *GAA* variants and confirmation of Pompe disease

The detected variants were matched to the patient’s phenotype and those that were most likely to be disease-causing were reported back to the referring clinician. A positive diagnosis of Pompe disease was sought by the clinicians by quantifying α-glucosidase activity using dried blood spots (DBS) or fibroblasts, and/or performing Sanger sequencing to independently confirm the detected variants.

## Results

### Detection of variants within *GAA*

Of the 606 patients whose exomes were sequenced, 268 (44%) were female and 338 (56%) were male; their ages ranged from 4 years to 88 years (mean 40.1 years, median 40.0 years). In the first instance, a search of the *GAA* exons of all 606 patients was performed to identify those who harboured rare (< 1%) coding variants. A total of 34 distinct coding variants were identified (Table 1) in 35 unrelated individuals. Of these variants, ten were synonymous and so were not considered potentially disease-causing. Of the remaining 24 variants, 16 were missense, three were frameshift, one created a stop codon and four affected the splice site regions of the gene. Eight of the 24 variants were novel, meaning they did not occur in the ExAC v3 control population of over 60,000 unrelated healthy individuals [14]. Finally, six of the 24 variants, three of which were novel, have been previously listed in the Pompe Disease Mutation Database [6] and so have a known association with the disease.

**Table 1:** *GAA* variants detected in the patients sequenced by the MYO-SEQ project

Patient	Location			Reported	Polymorphism	Variant	Predicted deleteriousness				ClinVar clinical significance	ExAC v3 allele frequency
	Chromosome	Coding	Protein				SIFT	PolyPhen-2	MutationTaster2	FATHMM		
10	chr17:78,078,411	c.26C > G	p.Ser9Cys	No	–	Missense	Damaging	Benign	Polymorphism	Damaging	No data	0.00000
13	chr17:78,078,484	c.99 T > C	–	No	rs144736309	Synonymous	No data	No data	No data	No data	No data	0.00002
2	chr17:78,078,671	c.286A > G	p.Lys96Glu	No	–	Missense	Tolerated	Benign	Polymorphism	Tolerated	No data	0.00001
28	chr17:78,078,805	c.420C > A	p.Asn140Lys	No	–	Missense	Tolerated	Benign	Polymorphism	Damaging	No data	0.00002
18 and 32*	chr17:78,078,909	c.525delT	p.Glu176ArgfsTer45	Yes	–	Frameshift	No data	No data	No data	No data	Pathogenic	0.00007
9	chr17:78,078,930	c.545C > G	p.Thr182Arg	No	rs200524747	Missense	Tolerated	Benign	Disease-causing	Tolerated	No data	0.00006
35*	chr17:78,079,570	c.569G > A	p.Arg190His	Yes	–	Missense	Deleterious	Probably damaging	Disease-causing	Damaging	Likely pathogenic	0.00001
31	chr17:78,079,591	c.590C > A	p.Thr197Asn	No	–	Missense	Tolerated	Benign	Polymorphism	Damaging	No data	0.00001
24	chr17:78,079,677	c.676C > G	p.Leu226Val	No	rs113085339	Missense	Tolerated	Benign	Polymorphism	Damaging	Likely benign; uncertain significance	0.00070
15	chr17:78,081,352	c.693-4G > T	–	No	rs200088236	Splice region	No data	No data	No data	No data	No data	0.00031
30	chr17:78,081,518	c.855C > G	–	No	–	Synonymous	No data	No data	No data	No data	No data	0.00000
14	chr17:78,081,653	c.913G > A	p.Gly305Arg	No	rs200154987	Missense	Deleterious	Probably damaging	Disease-causing	Damaging	No data	0.00025
12 and 34	chr17:78,081,655	c.915G > A	–	Yes	rs150343359	Synonymous	No data	No data	No data	No data	Uncertain significance	0.00113
16	chr17:78,082,117	c.984 T > C	–	No	–	Synonymous	No data	No data	No data	No data	No data	0.00000
6	chr17:78,082,180	c.1047C > T	–	No	rs138262940	Synonymous	No data	No data	No data	No data	No data	0.00002
17*	chr17:78,082,399	c.1192delC	p.Leu398TrpfsTer42	No	–	Frameshift	No data	No data	No data	No data	No data	0.00000
26	chr17:78,083,737	c.1327-7 T > G	–	No	–	Splice region	No data	No data	No data	No data	No data	0.00001
21	chr17:78,083,769	c.1352C > G	p.Pro451Arg	No	rs7215458	Missense	Tolerated	Possibly damaging	Disease-causing	Damaging	No data	0.00043
21	chr17:78,084,516	c.1438-7_1438-5delTGT	–	No	–	Splice region	No data	No data	No data	No data	No data	0.00002
25	chr17:78,085,800	c.1655 T > C	p.Leu552Pro	Yes	–	Missense	Deleterious	Probably damaging	Disease-causing	Damaging	No data	0.00002
20 and 29	chr17:78,086,452	c.1830C > T	–	Yes	rs61736896	Synonymous	No data	No data	No data	No data	No data	0.00151
4 and 27	chr17:78,086,706	c.1920 T > G	–	Yes	rs144090460	Synonymous	No data	No data	No data	No data	No data	0.00032
35*	chr17:78,086,806	c.2020C > G	p.His674Asp	No	–	Missense	Deleterious	Probably damaging	Disease-causing	Damaging	No data	0.00000
8*	chr17:78,087,027	c.2051C > G	p.Pro684Arg	No	–	Missense	Deleterious	Probably damaging	Disease-causing	Damaging	No data	0.00000
5*	chr17:78,087,039	c.2066_2070dupAGCCG	p.Ala691SerfsTer7	Yes	–	Frameshift	No data	No data	No data	No data	No data	0.00000
19	chr17:78,087,046	c.2070G > A	–	No	–	Synonymous	No data	No data	No data	No data	No data	0.00003
33	chr17:78,087,131	c.2155G > A	p.Ala719Thr	No	–	Missense	Tolerated	Benign	Polymorphism	Damaging	No data	0.00004
1	chr17:78,087,133	c.2157G > A	–	No	rs201523530	Synonymous	No data	No data	No data	No data	Uncertain significance	0.00014
7*	chr17:78,090,846	c.2269C > T	p.Gln757Ter	Yes	rs200483245	Stop gained	No data	No data	No data	No data	No data	0.00000
23	chr17:78,090,907	c.2330C > T	p.Thr777Met	No	–	Missense	Tolerated	Benign	Polymorphism	Damaging	No data	0.00002
3*	chr17:78,090,910	c.2331 + 2 T > A	–	Yes	–	Splice donor	No data	No data	No data	No data	No data	0.00000
11	chr17:78,091,525	c.2458G > T	p.Ala820Ser	No	–	Missense	Tolerated	Benign	Polymorphism	Damaging	No data	0.00002
17*	chr17:78,092,521	c.2716G > A	p.Val906Ile	No	–	Missense	Tolerated	Benign	Disease-causing	Damaging	No data	0.00000
22	chr17:78,092,562	c.2757C > T	–	No	–	Synonymous	No data	No data	No data	No data	No data	0.00005
3, 5, 7, 8, 18, 32	chr17:78,078,341	c.-32-13 T > G	–	Yes	–	Intronic	No data	No data	No data	No data	No data	0.00360

Rows 1-34: all rare (< 1%) coding variants detected. Those highlighted with an asterisk (*) were classified as disease-causing and occurred in combination with the c.-32-13 T > G intronic variant for all but patients 17 and 35. Row 35: intronic c.-32-13 T > G transversion considered to contribute to disease pathology. Reported variants are listed in the Pompe Disease Mutation Database [6]

Since there were no homozygous coding variants that could in isolation account for the phenotype of the patients, and in fact an extremely common *GAA* mutation in Pompe disease is intronic [23], we extended the search to include flanking regions that were captured by the exome sequencing. As a result, the common intronic c.-32-13 T > G variant was detected and was considered potentially pathogenic when in combination with either a coding or an already reported pathogenic variant (Table 1).

### Analysis of *GAA* variants and classification of disease-causing mutations

**Fig. 1:**
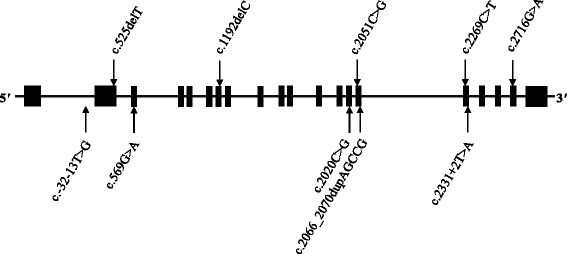
Ten distinct variants within *GAA* were identified as disease-causing. One variant was intronic and nine were exonic. Six patients were heterozygous for the intronic c.-32-13 T > G variant in addition to an exonic variant and two patients were heterozygous for two exonic variants

It was next necessary to interrogate the 22 patients that accounted for the 24 coding and one intronic *GAA* variants that were potentially disease-causing. Patients 3, 5, 7, 8, 18, and 32 all harboured the intronic c.-32-13 T > G variant in addition to a coding variant within *GAA*, which generated a compound heterozygous haplotype that could be responsible for α-glucosidase deficiency. Patients 17 and 35 carried two coding variants: patient 17 carried one frameshift and one missense, both of which were absent in the ExAC control population [14], and patient 35 harboured two missense variants, one of which was absent in the ExAC population [14] and the other previously reported in the Pompe Disease Mutation Database [6]. Patient 21 harboured a missense variant that was predicted to be damaging, while a second variant affected a splice site, neither of which were novel: this was not considered a likely cause of the patient’s phenotype. The ten variants considered disease-causing are detailed in Fig. 1, and the summarised patient information is detailed in Table 2. The remaining 14 patients only carried one heterozygous variant, meaning it was highly probable that α-glucosidase deficiency was not the cause of their disorder.

**Table 2:** Demographic information and reported clinical presentations of eight patients with causal variants in *GAA*

Patient	Sex	Ethnicity	Variant 1	Variant 2	Pace of progression	Onset	Serum creatine kinase	Weakness	Respiratory insufficiency	Referred for treatment
3	Male	German	c.2331 + 2 T > A	c.-32-13 T > G	Slow progression	Young adult	Increased less than 10×	Yes	No	Yes
5	Male	Romanian	c.2066_2070dupAGCCG	c.-32-13 T > G	Slow progression	Middle age	Increased more than 10×	Yes	No	Yes
7	Female	Serbian	c.2269C > T	c.-32-13 T > G	Slow progression	Middle age	Increased less than 10×	Yes	No	No
8	Female	Serbian	c.2051C > G	c.-32-13 T > G	Slow progression	Late onset	Normal	Yes	No	No
17	Male	Caucasian	c.1192delC	c.2716G > A	Progressive	Young adult	Normal	Yes	Yes	Yes (now ceased)
18	Female	White British	c.525delT	c.-32-13 T > G	Slow progression	Middle age	Increased more than 10×	Yes	No	Yes
32	Male	Caucasian	c.525delT	c.-32-13 T > G	Non-progressive	Childhood	Increased less than 10×	No	No	Yes
35	Male	Caucasian	c.569G > A	c.2020C > G	Progressive	Young adult	Increased less than 10×	Yes	No	Yes

### *GAA* variants were associated with a highly varied clinical spectrum

There was a wide variability in the presenting symptoms between each of the eight patients, despite the variants all affecting the same gene. This was particularly notable for patients 18 and 32 who both carried the same *GAA* variants, c.525delT and c.-32-13 T > G. Patient 18 had a slowly progressive phenotype after the onset of symptoms in her fifth decade of life, displaying proximal muscle weakness and a serum creatine kinase activity of 599 U/L (*N* < 170 U/L). The patient also showed a myopathic electromyogram (EMG). A muscle biopsy was indicative of a myofibrillar myopathy, with numerous vacuolated, degenerated and atrophic fibres confirmed by NADH-tetrazolium reductase staining. The periphery of the vacuoles had an abnormal immunoreactivity for desmin and p62. The fibre type proportions and distributions appeared normal; however an ultrastructural examination displayed Z-band streaming with an accumulation of granular material. Glycogen accumulation in the vacuoles was noted, but the overall appearance was not suggestive of a glycogenosis, while a DBS test revealed α-glucosidase activity to be within the normal to lower ranges. Following our report of the *GAA* variants to the collaborating centre, this patient has since been referred for ERT.

In contrast, patient 32 had a non-progressive phenotype after the onset of his symptoms in childhood. His serum creatine kinase activity was persistently elevated; when first investigated at age 15 years of age it was 662 U/L (*N* < 190 U/L). The patient had no cardiac involvement nor exhibited extreme respiratory distress, although a 13% reduction between sitting and supine forced vital capacity was observed. The patient described only slight dyspnoea following physical exertion, but otherwise considered himself healthy. Both a muscle biopsy and an EMG were mildly myopathic. A DBS test displayed reduced enzymatic activity, while Sanger sequencing of the variants confirmed Pompe disease. Additionally, the reduction in α-glucosidase activity and carriage of the variants were confirmed in his two siblings. Both, however, are currently asymptomatic with elevated serum creatine kinase levels only (563 U/L and 1331 U/L for his 10 year old sister and 23 year old brother, respectively). ERT is scheduled for the index case, and all three of these family members will be closely monitored.

A further example to highlight the varied clinical presentations was seen in patient 3. This individual displayed a slowly progressive proximal lower limb and axial weakness with the onset of symptoms in his third decade of life. There was no respiratory insufficiency and an EMG showed no abnormalities. Despite normal cardiac function, the patient was previously diagnosed with Brugada syndrome, a genetic cause for abnormal electrocardiogram findings that are associated with an increased risk of sudden cardiac death. Serum creatine kinase activity was measured at 1729 U/L (*N* < 190 U/L). The two *GAA* variants were confirmed by the collaborating centre using Sanger sequencing, while a reduced enzymatic activity was confirmed using a DBS test. Since the diagnosis of Pompe disease in the index case, he was started on ERT and his asymptomatic brother, with only an elevated serum creatine kinase activity, was also diagnosed with the disease. This clearly demonstrates that related individuals diagnosed with the same genetic disease can present with a varied phenotypic spectrum.

Patient 5 initially presented with back pain and elevated serum creatine kinase levels (1500 U/L), and retrospectively recognised mild proximal upper limb weakness when raising a load. The disease course progressed over the following nine years, with asymmetrical proximal weakness becoming more obvious. At hospitalisation, a reduced enzyme activity was observed in the patient and also in his daughter. Only two patients were not started on ERT: patient 7 lives in another country to where she was enrolled onto the project and patient 8 is in her mid-70s and is not ambulant.

### Comparison of compound heterozygous patients at two exonic *GAA* variants

Patient 17 harboured two novel coding variants within *GAA*, c.1192delC and c.2716G > A, neither of which have been listed in the Pompe Disease Mutation Database [6]. Importantly, however, c.1192delC results in a frameshift mutation at the same position as a reported pathogenic duplication [24]. The patient presented with progressive proximal muscle weakness, pain and fatigability in his fourth decade of life, but with normal levels of serum creatine kinase activity. He also displayed weakness of the back muscles and had notable respiratory dysfunction: his forced vital capacity was reduced to 2.41 (47% predicted normal value) in the sitting and to 2.0 l (39% predicted normal value) in the lying position. An EMG detected mild myopathic changes and the muscle biopsy showed increased variability in fibre diameter without any abnormal glycogen accumulation. The patient had two DBS tests: the first suggested a reduction in α-glucosidase activity, while the second detected normal levels of enzyme activity. A subsequent analysis of α-glucosidase in lymphocytes and fibroblasts revealed borderline and low normal activity, respectively. It is likely, therefore, that c.2716G > A may be a weaker mutation, and so only slightly affects enzyme activity. The variants were independently confirmed by the collaborating centre before ERT was initiated for the patient. At the start of ERT, the patient achieved 100 m in a six minute walk test, which marginally increased to 105 m after 9 months of alternating weekly administrations of 20 mg/kg Myozyme®. Measures of respiratory muscle strength similarly remained unchanged; from a vital capacity of 45% to 47%, a maximal inspiratory pressure of 5.6 kPa to 5.8 kPa and a maximal expiratory pressure of 6.1 kPa to 5.4 kPa. The referring clinician observed a negligible effect on muscle pain and weakness – as might be expected with borderline enzymatic activities treated over such a short period – and so the patient was withdrawn from the regime. Eighteen months after ERT cessation, the status of the patient remained stable with a six minute walk test of 95 m.

Similarly, patient 35 carried two coding variants within *GAA*: c.569G > A and c.2020C > G, with the former recognised in the Pompe Disease Mutation Database [6] and the latter completely absent in the ExAC unaffected control population [14]. The patient presented in his fourth decade of life with rapidly progressive proximal lower limb weakness, difficulty in ascending stairs and an inability stand unaided from a supine position. His older sister is also affected with an earlier onset of her symptoms at 21 years of age. The patient now has scoliosis, fatigability, paraspinal muscle atrophy, and proximal upper limb atrophy and weakness. Paraclinical examinations revealed a myopathic EMG, a dystrophic biopsy and mildly elevated serum creatine kinase levels. A calpain deficiency was detected through immunoblot analysis and could be attributed to two heterozygous *CAPN3* variants (p.Gly333Asp [c.998G > A] and p.Ala726Ser [c.2176G > T]). This suggested that the patient is likely to be affected by both LGMD2A and Pompe disease. The two *GAA* variants were independently confirmed and in contrast to patient 17, the unambiguous absence of glycogen was confirmed in the patient and in his sister. ERT is now being administered to both individuals.

## Discussion

Whole exome sequencing is emerging as an affordable technology to investigate rare, monogenic diseases. Coding and functional regions account for only 1% of the entire human genome, yet harbour 85% of known disease-causing variants [25]. On this basis, we sequenced the exomes of a cohort of 606 patients with unexplained limb-girdle weakness. We examined a panel of 169 genes that were known to be associated with muscle diseases with Mendelian patterns of inheritance in a large cohort of European patients with unexplained limb-girdle weakness. Overall, we identified eight unrelated index cases and four siblings that had compound heterozygous mutations in *GAA*: these patients were likely to be affected by Pompe disease, a rare lysosomal storage disorder that is commonly characterised by proximal muscle weakness [2]. The remaining 168 genes are currently under analysis and we are yielding similarly positive results for many other muscle diseases; so far the overall diagnostic rate for the project is 49%.

We have shown that next-generation sequencing is advantageous in the healthcare and diagnosis of patients suffering from unexplained limb-girdle muscle weakness. Other studies have shown similar benefits [26], achieving a correct diagnosis for patients with overlapping, and therefore indistinguishable, clinical phenotypes [27]. In a recent publication that interrogated the exomes of 504 patients of European descent, variants in *GAA* were considered pathogenic in 10 patients (1.9% of the cohort) [28]. This is highly comparable to the 12 patients with *GAA* variants detected by MYO-SEQ. While such an approach helps better understand rare neuromuscular disorders, it was especially important in this study for the patients that suffered from treatable conditions such as Pompe disease. It is most beneficial to the affected individuals that ERT is initiated as soon as possible as the therapy acts to ameliorate disease progression [29]. Despite DBS tests being widely available, inexpensive and sensitive to changes specifically in α-glucosidase activity [30], they can sometimes fail to robustly detect subtle changes in enzyme activity. This is considered more common in later onset forms of the disorder, where the level of enzyme activity may be proportional to the age of onset [29]. Although reduced enzymatic activity was detected by DBS tests in 7.6% of patients in a cohort presenting with elevated serum creatine kinase and/or limb-girdle muscular weakness, late-onset Pompe disease was only confirmed in 2.4% of the patients [31]. DBS tests may therefore be inconclusive for many patients with a proximal muscle weakness phenotype. Such a discrepancy was exemplified in the clinical assessment of patient 18 who presented with the onset of symptoms in her fifth decade of life and normal to slightly lower α-glucosidase activity. Despite the borderline enzymatic activity levels detected, the individual was subsequently found to carry two reported pathogenic *GAA* variants. Therefore, exome sequencing offers an alternative, accurate and reliable methodology for rare disease diagnosis where traditional detection techniques may not be as efficient. We suggest that DBS should still be used as a first-tier diagnostic step when Pompe disease is suspected, however, diagnostic yields are influenced by the type and level of pre-screening, and so it is inaccurate to compare the outcomes of different approaches.

As Pompe disease is an autosomal recessive disorder, single heterozygous variants are unlikely to result in a depletion of α-glucosidase activity that is sufficient to cause a clinical phenotype. Accordingly, it has been found that many affected individuals carry compound heterozygous mutations rather than isolated heterozygous or homozygous variants [32–35]. This immediately allowed our analysis to be focussed on the patients that harboured two variants: of course, a second variant may still be missed if it resides in a deep intronic region not covered by WES. Nevertheless, of the nine compound heterozygous patients that we identified, we considered eight to carry variants that were sufficiently severe to cause Pompe disease. Four of the ten likely pathogenic variants that were identified in this study had never been previously reported in Pompe disease [6] and did not occur in the ExAC control population [14]. These variants that we present here – c.1192delC, c.2020C > G, c.2051C > G and c.2716G > A – are therefore novel in the understanding of Pompe disease.

As would be expected based on previous findings [23, 35], the intronic c.-32-13 T > G variant was the most frequent in this largely Caucasian population, and occurred in six compound heterozygous index cases. It is therefore essential that analyses of *GAA* gene sequences should be extended to flanking regions in order to capture such pathogenic intronic variants. This variant affects a splice site of the gene, and so the result is the production of alternative transcript isoforms and low levels of α-glucosidase activity. This can give rise to the typical spectrum of Pompe disease phenotypes depending on the haplotype it occurs in [36]. Overall, the specific mutations, their haplotypes, and their genetic positions are all key in determining enzymatic activity and thus, the clinical phenotype of patients affected by Pompe disease.

## Conclusions

In summary, we have identified twelve individuals (eight index cases and four siblings) with compound heterozygous mutations in the *GAA* gene; four of the ten variants have not been previously reported. This study has expanded the existing genotype-phenotype correlations; aiding a deeper understanding of Pompe disease, the underlying genetic variations and the associated varied clinical presentation. We have shown the advantage of using next-generation sequencing in the diagnosis of a rare, treatable neuromuscular condition. As a result, patients have benefitted from a swifter administration of appropriate disease managements. Our data suggest that exome sequencing is a reliable and accurate diagnostic tool and is able to detect pathogenic variants in patients for whom traditional clinical methodologies have failed.

## Abbreviations

DBS: dried blood spot
EMG: electromyogram
ERT: enzyme replacement therapy
ExAC: Exome Aggregation Consortium
*GAA*: α-glucosidase encoding gene
min: minute
ml: millilitre
rpm: revolutions per minute
